# COGNITIVE PERFORMANCE, EXERCISE, AND AMYLOID BURDEN: A RANDOMIZED CONTROLLED TRIAL

**DOI:** 10.1093/geroni/igz038.2272

**Published:** 2019-11-08

**Authors:** Amber Watts, Eric Vidoni, Jill Morris, Mark Perry, David Johnson, Jeffrey Burns

**Affiliations:** 1 University of Kansas, Lawrence, Kansas, United States; 2 University of Kansas Medical Center, Kansas City, Kansas, United States; 3 UC Davis, Davis, California, United States

## Abstract

Exercise is a promising strategy for prevention of Alzheimer’s disease (AD). Amyloid neuroimaging can identify individuals at risk of developing AD prior to displaying symptoms. We screened adults (65+) with Florbetapir PET imaging and a comprehensive cognitive battery. We randomized 117 participants with normal cognition into a 52-week aerobic exercise program to examine the effects of aerobic exercise on cognitive performance. We compared an intensive exercise treatment group to a standard of care control group. Cognition was assessed at baseline, 26 weeks, and 52 weeks in the domains of verbal memory, visuospatial processing, attention, and executive function. Interim results on 87 participants show cardiorespiratory fitness improved in the exercise group vs. control group (t=3.66(81), p< .001). The degree of change in cardiorespiratory fitness did not differ between those with and without elevated amyloid (t=-0.37(81), p=.710). Greater improvements in cardiorespiratory fitness predicted better performance on cognitive tests including trailmaking test, Stroop test, and digit symbol substitution test, which did not differ by amyloid status. Elevated amyloid levels predicted lower cognitive scores in logical memory, space relations, and identical pictures test. Our findings suggest PET imaging is a valid marker of cognitive performance in non-impaired older adults, and that this pre-clinical amyloid status did not reduce the cognitive benefits of exercise for those who improved in cardiorespiratory fitness. Exercise interventions hold promise for cognitive maintenance among pre-symptomatic older adults with elevated amyloid levels. Finally, results highlight the importance of evaluating multiple cognitive domains which are associated differently with exercise and amyloid status.

